# The effect of canopy exchange on input of base cations in a subalpine spruce plantation during the growth season

**DOI:** 10.1038/s41598-018-27675-9

**Published:** 2018-06-19

**Authors:** Siyi Tan, Hairong Zhao, Wanqin Yang, Bo Tan, Xiangyin Ni, Kai Yue, Yu Zhang, Fuzhong Wu

**Affiliations:** Long-term Research Station of Alpine Forest Ecosystems, Key Laboratory of Ecological Forestry Engineering, Institute of Ecology and Forestry, Sichuan Agricultural University, Chengdu, 611130 China

## Abstract

Canopy exchange is one of the most important processes involved in the internal transfer of elements in forest ecosystems. However, little information is available on how canopy exchange influences the input of base cations in subalpine forests. Therefore, the concentrations and fluxes of base cations in throughfall and stemflow were investigated from August 2015 to July 2016 (except for the frozen season) in a representative subalpine spruce plantation in the eastern Tibet Plateau. Our results showed that the mean concentrations of K, Ca, Na and Mg were higher in the stemflow than in the throughfall and precipitation. The total input fluxes of K, Ca, Na and Mg in the internal forest were lower than those in the non-forest. Moreover, the results from the canopy budget model indicated that the canopy exchange fluxes of K, Ca and Mg were higher than the dry deposition fluxes, and Ca and Mg were uptaken, whereas K was leached when precipitation passed through the canopy. Therefore, the results suggested that the input of base cations is mainly controlled by canopy exchange during precipitation in subalpine forest ecosystems, and the canopy could alter the sinks and sources of base cations from precipitation.

## Introduction

Precipitation often acts as a primary transporting agent and solvent; thus, element cycles in forests are always closely linked to the hydrological process^1^. The transfer of elements via precipitation deposition has a critical impact on the biogeochemistry and bioavailability of nutrients^2^, especially the transfer of base cation elements (K, Ca, Na and Mg)^3^. Throughfall and stemflow are two main types of precipitation transport in forest ecosystems. Throughfall reaches the ground by passing directly through or dripping from the vegetation canopy, which provides a crucial cycling medium by moving elements from the canopy to the soil and determines the biogeochemical cycles^4^. Stemflow delivers water to the ground via branches and tree stems^5,6^, and base cations are transported greater distances by stemflow than throughfall. Both types of water fluxes are important pathways in the internal element dynamics of forests and are essential to the growth of a forest community^7–9^. The elements that are transported via throughfall and stemflow are directly available to plants without the intervention of decomposition processes, which are required during the slow release of elements from litterfall^1^. Even so, the inputs of some elements such as K may be higher via throughfall than litterfall^10^. However, it is not clear how elements are transferred via precipitation, throughfall and stemflow since forest type and canopy characteristics could have profound effects on the transfer.

Forest canopies receive chemical inputs from the atmosphere as wet deposition and dry deposition^11,12^. The forest canopy can be subject to higher rates of dry deposition than non-forest areas^13^. In addition, the contents of elements such as K and N from precipitation can increase through the canopy, and the canopy can absorb pollution gasses such as SO_2_ and Cl_2_; hence, the presence or absence of vegetation is more important than the type of vegetation in a plantation forest^14^. The transfer of elements to the forest land is primarily via precipitation and is more influenced by the canopy^15^. The elements from precipitation that travel through the canopy are changed by two processes: (i) the washing of the dry deposition accumulated on the canopy, and (ii) the canopy exchange process through the leaching and uptake from the canopy^2^. However, it is difficult and necessary to distinguish dry deposition from canopy exchange because dry deposition is an important mechanism of atmospheric inputs to forested ecosystems, and canopy exchange is the transfer elements within an ecosystem^16,17^. Therefore, a canopy budget model can be used to adequately identify and evaluate dry deposition and canopy exchange^4,18^. This model is based on the assumptions that Na is deposited by only particles and the Na canopy leaching rates are low. Thus, Na can be used as a tracer ion to evaluate the fluxes of dry deposition and canopy exchange of K, Ca and Mg^11^.

Over the past several decades, a large number of pure spruce plantations have replaced the natural coniferous forests on the Tibetan Plateau that have been harvested by large-scale industrial logging operations^19^, which play an important regulatory role in regional climate and water conservation^20^. The local knowledge of the variations in water chemistry is an important prerequisite to understanding the processes that occur within the forest stands. Therefore, many studies have evaluated the differences in the hydrology and chemical processes between throughfall and stemflow of different forests in different regions in China^12,21,22^. However, those studies did not focus on the role of canopy exchange processes in forest ecosystems, and research on hydrology in subalpine forests is scarce. Therefore, this study presents the results of an analysis of rainfall, throughfall and stemflow data collected during the growth season in spruce plantations. The objectives of the research are to (i) observe the variations in the base cations and, (ii) evaluate the dry deposition and canopy leaching or uptake fluxes of base cations in the spruce canopy. This research can help understand how the coniferous forest canopy can alter the elements of precipitation processes and provide basic data for subsequent research in alpine forest ecosystems.

## Results

### The variations in base cation concentrations

**Figure 1:**
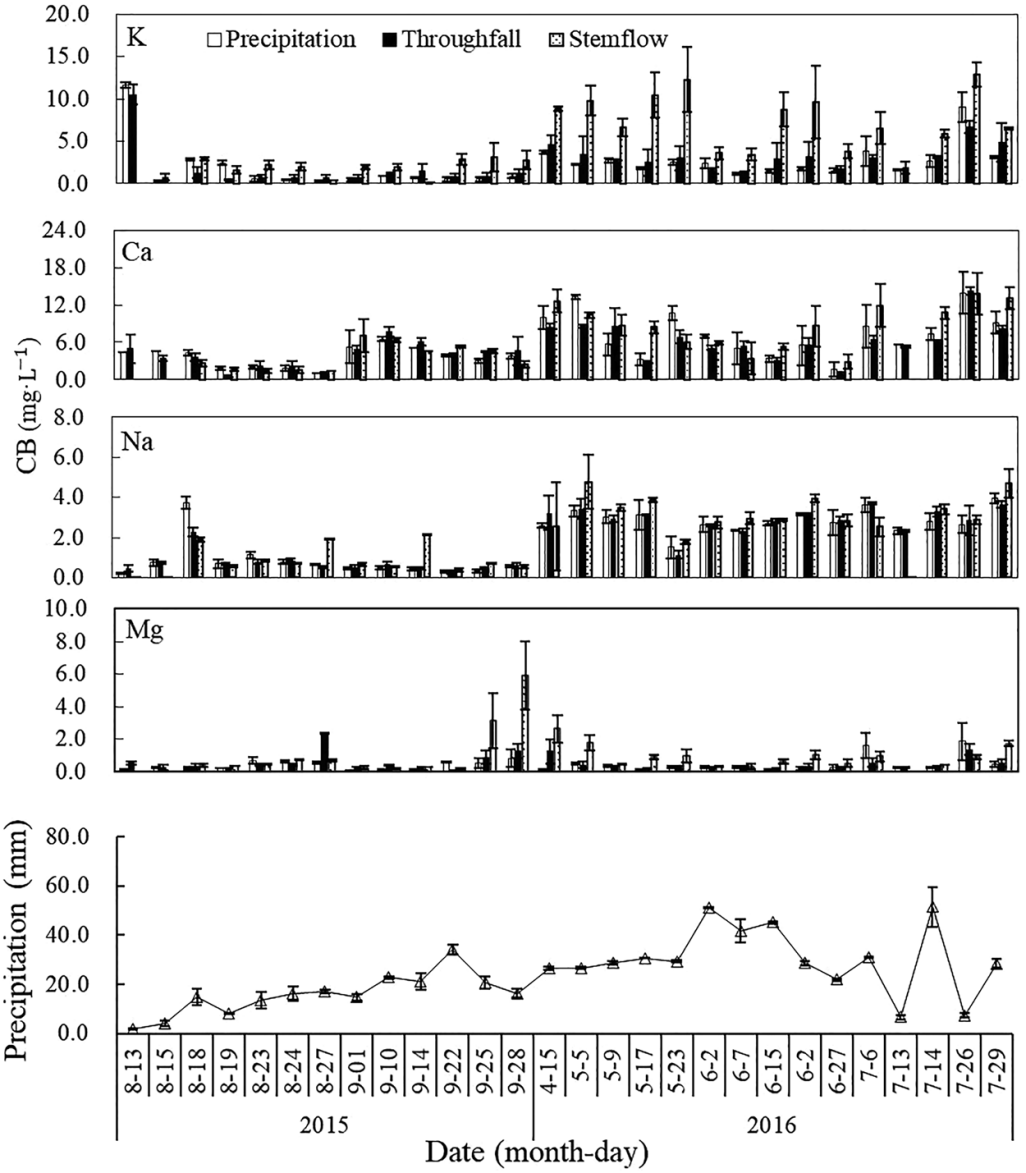
The volumes of precipitation and the concentrations of base cations (CB) of rainfall, throughfall and stemflow variations. The bar graphs and line graph with error bars are the means with 95% confidence intervals.

The total concentrations of all base cations exhibited the order of stemflow > throughfall > precipitation (Fig. 1). The concentrations of K, Ca, Na and Mg were from 0.51 to 5.72 mg·L^−1^. The concentration of Ca dominated among the base cations in the three types of water, followed by the concentrations of K, Na and Mg. K exhibited the largest variation among the base cations in the stemflow, with a variation that was 1.93-fold and 2.05-fold higher than the variations in the throughfall and precipitation, respectively. In addition, the concentrations were positively correlated with throughfall, and the correlations showed an increase in the concentrations of base cations with the increase in throughfall (except Mg), as shown in Table 1. Moreover, the relationship between the concentrations of K and the volume of throughfall was strong, with an R^2^ value of 0.717. However, there was no relationship between the concentrations of base cations and the volumes of stemflow.

**Table 1:** The relationships between the concentration of base cations and volume of throughfall.

Base cations	Fitting equation	Parameters		n	R^2^	P
		a	b			
K	Y = aX + b	0.033	−0.259	28	0.717	**0.000**
Ca	Y = aX + b	0.057	−0.074	28	0.703	**0.000**
Na	Y = aX + b	0.030	−0.177	28	0.668	**0.000**
Mg	Y = aX + b	0.020	0.043	28	0.071	0.092

Bold values indicate the significant portion of the variation among effect concentration that can be explained by the volume of throughfall variable when *p* < 0.05.

### The variations in base cation input fluxes

**Figure 2:**
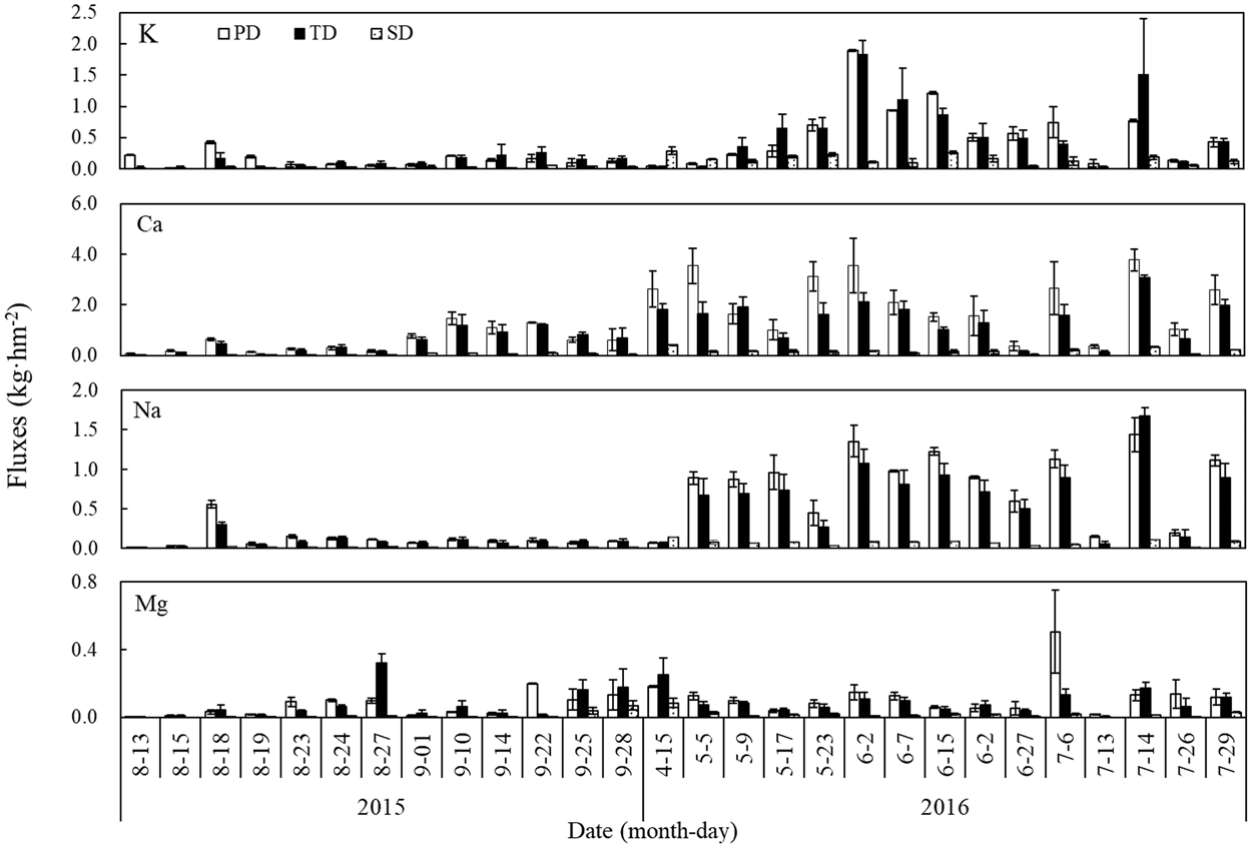
The variations in deposition fluxes of base cations shown as precipitation deposition (PD), throughfall deposition (TD), and stemflow deposition (SD). The bar graphs with error bars are the means with 95% confidence intervals.

The total inputs of base cations in precipitation deposition, throughfall deposition and stemflow deposition at the study site were 66.20, 52.50 and 7.00 kg·hm^−2^, respectively (Fig. 2). The total deposition of base cations was 1.11 times higher in the non-forest than in the closed canopy. During the study, the throughfall deposition and stemflow deposition of base cations accounted for 88.24% and 11.76% of the total input, respectively. The total input fluxes of K, Ca, Na and Mg were 13.03 kg·hm^−2^, 31.30 kg·hm^−2^, 12.40 kg·hm^−2^, and 2.77 kg·hm^−2^, and those in the non-forest were 10.50 kg·hm^−2^, 39.10 kg·hm^−2^, 13.91 kg·hm^−2^, and 2.75 kg·hm^−2^, respectively.

**Figure 3:**
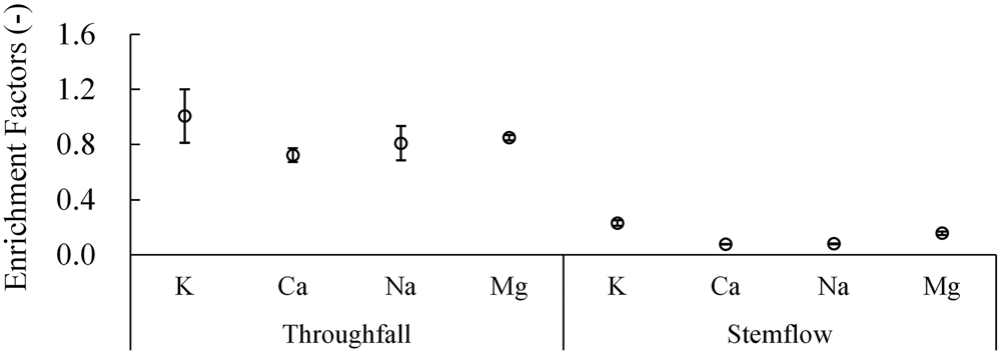
The enrichment factors of base cations for throughfall and stemflow. The dots with error bars are the means with 95% confidence intervals.

The patterns in throughfall and stemflow are reflected in the enrichment factors of the base cations throughout the growth season (Fig. 3). The throughfall enrichment factor was 1 for K, and the smallest enrichment factor was reported for Ca. The enrichment factors for Na and Mg were intermediate. The base cation enrichment factors in stemflow were less than 1, and K had the largest enrichment factor among the base cations, followed by Mg, Na and Ca.

### Canopy exchange and dry deposition fluxes of base cations

**Figure 4:**
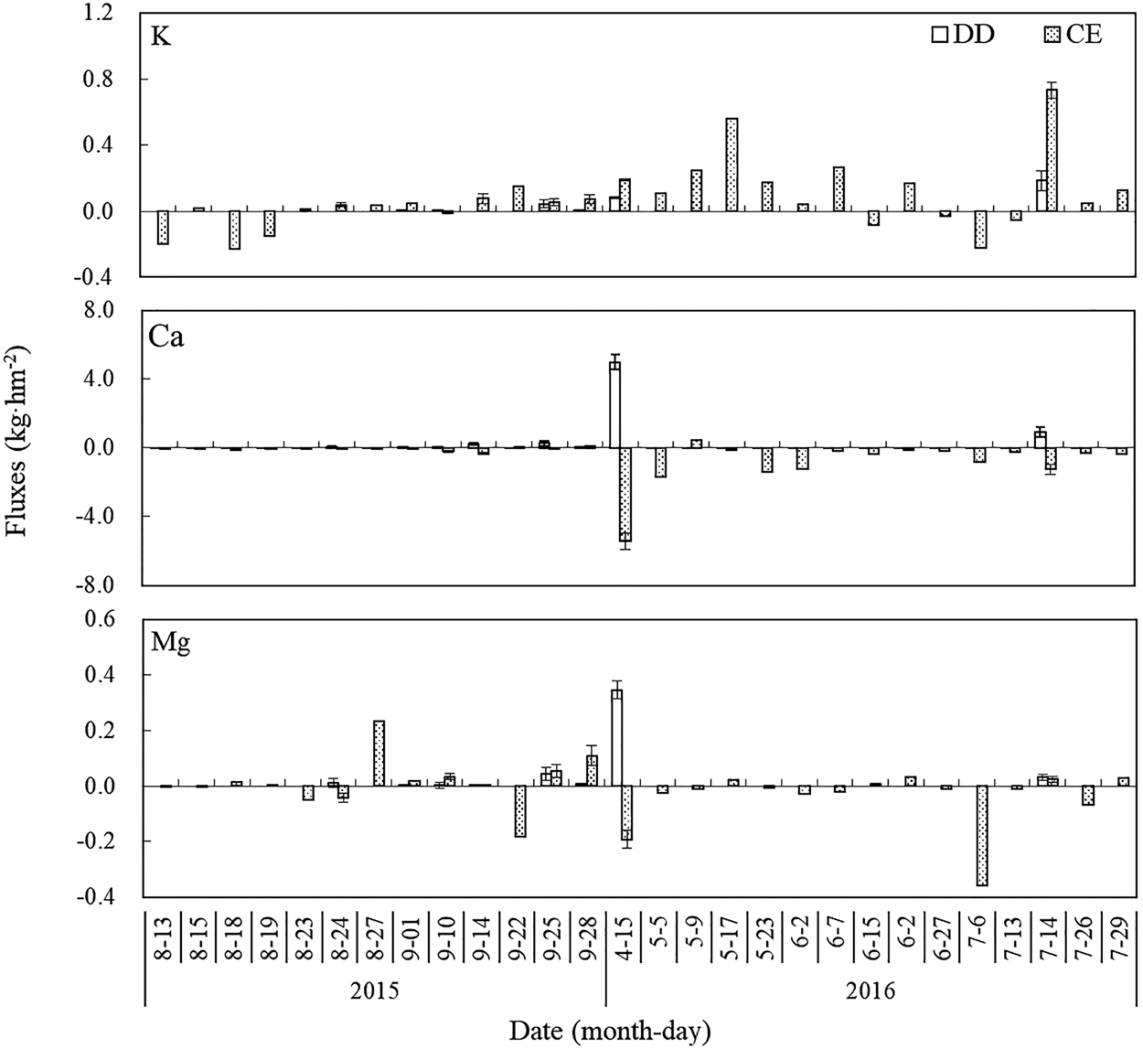
The canopy exchange (CE) and dry deposition (DD) fluxes during a single rainfall event in the growth season. The bar graphs with error bars are the means with 95% confidence intervals.

Figure 4 indicates that dry deposition alternates with canopy exchange of K, Ca and Mg during the growth season. Ca and K exhibited the highest and lowest dry deposition fluxes among the base cations. The dry deposition fluxes of K, Ca and Mg were 0.32 kg·hm^−2^, 6.49 kg·hm^−2^ and 0.44 kg·hm^−2^, respectively. The canopy exchange fluxes of Ca and Mg were most often negative, whereas they were positive for K, indicating that Ca and Mg were taken up by the canopy at rates of 14.35 kg·hm^−2^ and 0.43 kg·hm^−2^, respectively, and K was leached from the canopy at a rate of 2.20 kg·hm^−2^.

## Discussion

The interaction between rainfall and the canopy can change the contents of elements^23^. It is a common occurrence for the concentrations of elements to increase after precipitation passes through the canopy. The concentrations of base cations were 2.33 times higher in the closed forest canopy than the non-forest. The concentrations of elements followed the order of stemflow > throughfall. This order is due to the sufficiently long residence times of the intercepted precipitation on the branch surfaces, which increases the concentrations of elements, and the higher leachability of bark tissue for stemflow than throughfall^24^; this phenomenon also occurs in other forest ecosystems^25,26^. In addition, the concentrations of base cations were positively related to the volume of throughfall, except for Mg because the main source of Mg is dissolved in marine air. Therefore, Mg is less affected by the volume of water, and this result was also observed by Scheer^15^. The stemflow volume was not related to the concentrations of base cations. The bark surface and angle of the branches may have stronger effects on the concentrations of base cations than the volume of the stemflow.

In addition, the total inputs of base cations in the closed forest and non-forest were 59.50 kg·hm^−2^ and 66.20 kg·hm^−2^, respectively. The inputs of base cations from throughfall and stemflow accounted for 88.24% and 11.76% of the total input, respectively. The base cations fluxes were associated with the concentrations and volumes of water, and the stemflow volumes were lower than the throughfall volumes. Although the proportion of stemflow was lower than the proportion of throughfall, stemflow still plays an important role in the direct delivery of base cations to plant roots and the transport of nutrients over long distances, especially in ecosystems with sandy soils that are extremely nutrient-poor^27,28^.

A canopy budget model can be used to estimate the changes to base cations that occur within the forest canopy and distinguish between the internal (canopy exchange) and external input (dry deposition) sources to the ecosystems^29^. In this study, canopy exchange is much more common than dry deposition in sampling events for base cations, and canopy exchange is 2.33-fold higher than dry deposition. Therefore, the contribution of canopy exchange was higher than the contribution of dry deposition, especially during the growth season, and this result has also been found in other research^4,30^. There are two reasons for this result: first, the amount of precipitation is an important factor in canopy exchange^31,32^. The abundant rainfall during the growing season results in the saturation of the canopy and the leaching of more mobile elements along with the base cations. Second, the climate interacts with vegetation and can influence the deposition of elements^33^. The amount of dry deposition received by the canopy is lower in the winter than during the growth season, and canopy uptake and leaching are highest during the growth season in the closed canopy^34^.

**Figure 5:**
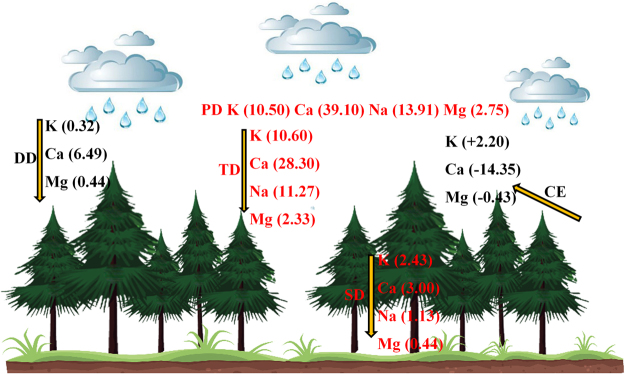
Precipitation deposition (PD), throughfall deposition (TD), dry deposition (DD) and canopy exchange (CE) processes in a subalpine forest (values are in kg·hm^−2^; − and + represent uptaken and leached, respectively).

The wash-off and leaching dynamics of throughfall ionic fluxes represent a significant process that affects the biogeochemical cycles of forest ecosystems^35^. The dry deposition and canopy leaching fluxes of Ca and, Mg are maximum in April. The canopy freeze-thaw effect could influence leaf physiology and further influence the canopy exchange fluxes of Ca and Mg in the early spring season. The dry deposition fluxes are mainly due to the greater interception of aerosols by the coniferous canopy, and the dry deposition that accumulates in the canopy during the dry (frozen) season is normally effectively washed at the beginning of rainfall episodes and is quickly exhausted^36,37^. The canopy exchange fluxes of K showed that the canopy leaches K in the spruce forest. In addition, the base cation enrichment factors of throughfall and stemflow were generally high for K, suggesting that K is the most leached of the base cations from the canopy and tree stem, leading to significant canopy exchange across various forest types^15,25^. In contrast, the enrichment factors of Ca and Mg were lower than 1, and the canopy exchange values of both elements were negative, indicating that Ca and Mg were uptaken by the canopy (Fig. 5). Identical results have also been found in other research^4,38,39^.

The canopy exchange is influenced by forest type and the concentration of solutes in precipitation, whereas dry deposition is influenced by drought time and exposure of the canopy layer to atmospheric deposition^40^, which results in different fluxes of both processes in different ecosystems^39,41,42^. Our results represent only a spruce plantation ecosystem in a subalpine forest. However, we consider the differences between the season variation and distinguished the growing season and the non-growth season and selected an appropriate sampling time (expect the frozen season), but interannual variability is still possible. Therefore, it is suggested that in the future long-term, a study should be set up, and repeating such measurements could strengthen the conclusions.

In summary, the interaction of precipitation with the canopy and tree stems causes a vertical spatial variation of the precipitation-throughfall-stemflow of base cations in forest ecosystems, which provides a pathway for base cations to move from the vegetation to the soil via throughfall and stemflow. In addition, the canopy budget model showed that the canopy exchange fluxes of K, Ca and Mg were higher than the dry deposition fluxes. The canopy exchange fluxes indicated that Ca and Mg were uptaken by the canopy, but K was leached. Therefore, canopy exchange is the main driver of the input of base cations in a spruce plantation during the growth season. The interannual variability of precipitation is possible, therefore, our study represents the variability of the chemistry of base cations during only our observation period. However, the knowledge of the distribution of elements in the canopy from precipitation in spruce plantation ecosystems is essential for understanding the circulation of forest elements and provide basic data for subsequent research in alpine forest ecosystems in future.

## Materials and Methods

### Study area

This study was conducted at the Long-term Research Station of Alpine Forest Ecosystems, Miyaluo Nature Reserve, Li County, Sichuan, southwest China, which is located along the eastern edge of the Tibetan Plateau, the western edge of the Sichuan Basin, and the upper Yangtze River^20^. The annual mean air temperature is 2–4 °C, and the maximum and minimum temperatures are 23.7 °C (July) and −18.1 °C (January), respectively. The mean annual precipitation is approximately 801~850 mm. Precipitation is mainly concentrated in the wet season (May~August) and the winter season from November to April^43^. The canopy forest vegetation is dominated by *Picea asperata* with some understory shrubs (e.g., *Salix paraplesia* and *Rhododendron spp*.) and grasses (e.g., *Berberis diaphana*, *Sorbus rufopilosa*, and *Deyeuxia scabrescens*), and the mean tree age is approximately 60 a.

### Study design

Three 400 m^2^ (20 m × 20 m) plots with similar locations, elevations, slopes, and other conditions were selected in a typical spruce forest (102°53′-102°57′ E, 31°14″-31°19′ N, 3000 m a.s.l.). It is difficult to find three non-forest near the spruce plantation forest, and we just only find one open area (20 m × 20 m) that was approximately 50 m from the edge of the spruce plantation forest which was selected as the non-forest site. The characteristics of the plots are shown in Table 2.

**Table 2:** Characteristics of vegetation in the plot.

	Canopy density	Mean DBH/cm	Mean height/m	basal areas/m^2^
Plot 1	0.6	21.7	8.1	0.0037
Plot 2	0.75	19.1	7.6	0.0029
Plot 3	0.8	17.8	7.2	0.0025

### Water sampling

There is not a strict distinction between the growing season and non-growing season. Therefore, we divided the growth season and non-growth season using the first snowfall and rainfall. Since the snowfall was heavy, and the field conditions were difficult, we set up the plots after the thawing period (May, 2015). To allow the device to adapt to the climate and environment in the area, the device underwent debugging for two months to prevent different efficiencies of the device. Thus, the water samples were collected after each rainfall event during the growth season from 13 August 2015 to 29 July 2016, and samples were not collected during the frozen season (November 2015 to March 2016). Torstenw and Li’s methods were used to collect the precipitation, throughfall and stemflow data, and the details are as follows^32,44^:

(i). Precipitation: the rainfall was sampled in the non-forest site with 5 homemade continuous rain gauges (surface collection area of 0.64 m^2^).
(ii). Throughfall: the throughfall was recorded using 5 PVC rectangle grooves (surface collection area of 400 cm × 16 cm) in the 3 plots, and 5 gutters were set up 1 m above the floor to avoid ground splash effects. Furthermore, the gutters were also set up at a 5° angle to horizontal to promote drainage, and the lower end of each gutter was equipped with a plastic bucket.
(iii). Stemflow: Before the sampling, we investigated the DBH, height and other characters, and found only several trees had DBH less than 15 cm and larger than 25 cm. We selected 9 trees with 10–15 cm, 15–20 cm, 20–25 cm classes in each plot. The trees were equipped with flexible tubing (plastic hose) that was cut in half lengthwise and fixed tightly around the stem. The tubing was approximately 1.3 m and was affixed in a steep upward spiral slope to avoid overflow. The tubing was attached to the tree trunk with nails, and silicone sealant was applied to seal the length of the tubing to the stem and avoid overflow, and the end of each section was connected to a polyethylene (PE) bottle. Stemflow did not occur following rain events less than 6.51 mm in this study.

### Data availability

All data generated or analysed during this study are included in this published article (and its Supplementary Information files).

### Chemical analyses

Water samples of up to 500 ml were collected in Nalgene polyethylene bottles that were prewashed with dilute (5%) HCl and then thoroughly rinsed with nanopure deionized water. The volumes of each reservoir were recorded. The samples were transported to the laboratory, where they were filtered using quantitative filter paper with a pore size of 0.45 μm and a diameter of 12.5 cm. The filtered samples were adjusted to a pH of 1~2 with high purity grade (GR) nitric acid and stored at 4 °C. The concentrations of K, Ca, Na, and Mg were measured using an atomic adsorption spectrophotometer (AA-7000, SHIMADZU, Japan).

### Calculations

Canopy budget model:

1 $${\rm{TD}}+{\rm{SD}}-{\rm{PD}}={\rm{DD}}+{\rm{CE}}$$

2 $${\rm{NTD}}={\rm{TD}}+{\rm{SD}}-{\rm{PD}}$$

3 $${\rm{DD}}={{\rm{PD}}}_{{\rm{x}}}\times {{\rm{NTD}}}_{{\rm{Na}}}/{{\rm{PD}}}_{{\rm{Na}}}$$

TD—throughfall deposition; SD—stemflow deposition; PD—open-field precipitation deposition DD— dry deposition; CE—canopy exchange; NTD—net throughfall deposition; X—(Ca, Mg, K). Canopy budget model^11,18,30^. The CE (Eq. 1) of K, Ca and Mg was then estimated by subtracting the DD (Eq. 3) from the NTD (Eq. 2), and when the NTD of the tracer ion (Na in this case) is negative, the DD is zero.

The enrichment factors of the base cations were calculated as the amounts of the base cations that were delivered as SD or TD divided by the PD^4^. Enrichment factors can reflect the extent of changes in the fluxes of ions from non-forest precipitation to water collected below the tree canopy in a forest.

### Statistical analysis

All statistical analyses were carried out using IBM SPSS statistics 20.0. The relationships between the concentrations of base cation in throughfall and stemflow were determined by using the regression analysis method. The statistical tests were considered significant at the *P* < 0.05 level.

## Electronic supplementary material

Dataset1
